# Direct Oral Anticoagulants in Cancer Patients. Time for a Change in Paradigm

**DOI:** 10.3390/cancers12051144

**Published:** 2020-05-02

**Authors:** Marek Z. Wojtukiewicz, Piotr Skalij, Piotr Tokajuk, Barbara Politynska, Anna M. Wojtukiewicz, Stephanie C. Tucker, Kenneth V. Honn

**Affiliations:** 1 Department of Oncology, Medical University of Białystok, 12 Ogrodowa St., 15-027 Białystok, Poland; skalij@piasta.pl (P.S.); ptokajuk@poczta.onet.pl (P.T.); 2 Department of Clinical Oncology, Comprehensive Cancer Center, 12 OgrodowaSt., 15-369 Białystok, Poland; 3 Department of Philosophy and Human Psychology, Medical University of Białystok, 37 Szpitalna St., 15-295 Białystok, Poland; bpolitynska@wp.pl (B.P.); aniawojtukiewicz@gmail.com (A.M.W.); 4 Robinson College, University of Cambridge, Cambridge CB3 9AN, UK; 5 Bioactive Lipids Research Program, Department of Pathology-School of Medicine, Detroit, MI 48202, USA; stucker@med.wayne.edu (S.C.T.); k.v.honn@wayne.edu (K.V.H.); 6 Department of Chemistry, Wayne State University, Detroit, MI 48202, USA; 7 Department of Oncology, Karmanos Cancer Institute, Detroit, MI 48202, USA

**Keywords:** thrombosis, cancer, treatment, prophylaxis, anticoagulants, DOAC

## Abstract

Thrombosis is a more common occurrence in cancer patients compared to the general population and is one of the main causes of death in these patients. Low molecular weight heparin (LMWH) has been the recognized standard treatment for more than a decade, both in cancer-related thrombosis and in its prevention. Direct oral anticoagulants (DOACs) are a new option for anticoagulation therapy. Recently published results of large randomized clinical trials have confirmed that DOAC may be a reasonable alternative to LMWH in cancer patients. The following review summarizes the current evidence on the safety and efficacy of DOAC in the treatment and prevention of cancer-related thrombosis. It also draws attention to the limitations of this group of drugs, knowledge of which will facilitate the selection of optimal therapy.

## 1. Introduction

As early as the nineteenth century, Armand Trousseau observed a relationship between the occurrence of venous thromboembolism (VTE) and cancer. Around one in five of all VTE cases are found in patients with oncological disease [1]. The risk of occurrence of VTE shows a four- to six-fold increase in patients with active oncological disease compared to the general population and is found in around 5–10% of patients with malignant disease. After progression of the malignant condition itself, it is the second most common cause of death in these patients [2]. Oncological treatment itself is an additional factor in raising the risk of thromboembolism. The co-occurrence of thrombosis and cancer creates many clinical problems. Firstly, VTE in patients with cancer worsens the prognosis by reducing objective survival rates. Secondly, the risk of recurrence of VTE and that of significant bleeding during the treatment of thrombosis is significantly increased in cancer patients compared to those without cancer (by three- and two-fold, respectively) [3]. In the absence of contradictions to its use, low molecular weight heparin (LMWH) remains the standard treatment for cancer-associated thrombosis (CAT), and LMWH has been confirmed to be superior to antagonists of vitamin K (VKA) in terms of efficacy and safety in many clinical trials [4,5,6,7]. Equally, a Cochrane meta-analysis demonstrated that LMWH reduces the risk of recurrent CAT without a significant increase in the risk of major or minor bleeding. This means that until recently LMWH has formed the backbone of most evidence-based recommendations for the treatment and prevention of venous thromboembolism in cancer patients, and many of these recommendations are still in operation [8,9,10]. Direct oral anticoagulants (DOAC), which include thrombin inhibitors (dabigatran) and factor Xa inhibitors (rivaroxaban, apixaban, and edoxaban) present a new option in the treatment and prevention of CAT. The specific properties of DOAC are particularly attractive due to the limitations of therapies in current use, such as the need for parenteral administration of LMWH as well as the need to monitor and modify the dose, and the high risk of interaction with food and other drugs in the case of VKA [11]. In the last two years, knowledge concerning the use of DOAC in cancer patients has significantly expanded. Is the emerging evidence sufficient to refute the current paradigm of the unrivaled role of LMWH in these patients? Below, the authors attempt to provide an answer to this question.

## 2. The Place of Direct Oral Anticoagulants in the Treatment of VTE in Patients with Cancer

To the best of the authors’ knowledge, no studies have, as yet, been specifically dedicated to directly comparing VKA and DOAC in the treatment of CAT in cancer patients. Large clinical trials carried out in members of the general population with a diagnosis of VTE have demonstrated the superiority of DOAC over VKA in terms of the effectiveness and safety of treatment, which has allowed the standards for management of patients without cancer to be modified accordingly, replacing warfarin with DOAC [12,13,14,15]. However, additional analyses of major clinical trials have also been performed. Selected sub-groups of cancer patients did not show significant differences between warfarin and DOAC with regard to the risk of VTE recurrence and major bleeding [16,17,18], defined as requiring transfusion or lowering hemoglobin by at least 2 g/dL [19]. However, it should be noted that the percentage of cancer patients in these studies was small. Additionally, the presence of variously-defined active cancers is likely to have been part of the exclusion criteria for the studies, and thus patients in the more advanced stages of the disease would not have been included. A meta-analysis of the results of patients meeting these exclusion criteria from six key phase III clinical studies demonstrated a statistically insignificant increase in the effectiveness and safety of DOAC as compared to VKA [20]. In turn, another meta-analysis covering the same sub-population of patients with cancer included in the studies described, showed a significant reduction in the risk of CAT recurrence among patients receiving DOAC as compared to patients in the control arm of the study receiving warfarin, while showing a similar risk of major bleeding [21].

The above data, suggesting the potential efficacy of DOAC in the treatment of CAT, prompted researchers to conduct randomized clinical trials directly comparing DOAC and LMWH in this condition. The results of four such studies have been published in the last two years. The first of them, Hokusai VTE Cancer, included 1050 cancer patients with newly diagnosed deep vein thrombosis, and symptomatic or accidental pulmonary embolism. Patients were given LMWH for five days and then continued on edoxaban (60 mg daily) or subcutaneous dalteparin (200 IU/kg/day for a month, then 150 IU/kg/day) for a period of 6–12 months. The primary efficacy endpoint was a composite factor that included recurrence of VTE or major bleeding. Edoxaban proved no worse than dalteparin (12.8% vs. 13.5%, respectively; *p* = 0.006 on noninferiority analysis). Statistically, the number of VTE recurrences was not significantly different between DOAC and LMWH treated groups (7.9% vs. 11.3%, *p* = 0.09), while major bleeding was more common in patients receiving edoxaban (6.9% vs. 4.0%, *p* = 0.04). No statistically significant differences were found in the number of clinically relevant non-major bleeding episodes (CRNMB). Of interest is the fact that attention was drawn to the high rate of CRNMB which reached 11.1% in the deltaparin arm in the Hokusai VTE Cancer clinical trial in which CRNMB was a secondary outcome. However, it is difficult to compare this result with the same parameter in other clinical trials due to the different protocol designs employed, distinct prespecified CRNMB definitions and different patient populations. Survival in both arms of the study was similar [22]. Another study (SELECT-D) compared the efficacy of rivaroxaban (given at a dose of 15 mg twice a day for three weeks, then 20 mg daily for six months) with dalteparin (200 IU/kg/day for a month, then 150 IU/kg/day for five months) in 406 patients with newly diagnosed CAT, defined as pulmonary embolism or deep vein thrombosis. The recurrence rate of VTE after six months was 4% in patients receiving rivaroxaban (95% CI 2–9%) and 11% (95% CI 7–17%) in patients receiving dalteparin. The number of major bleeds was similar in both arms of the study, 6% (95% CI 3–10%) in the rivaroxaban arm, and 4% in the dalteparin arm (95% CI 1–6%). However, there were differences in the number of CRNMBs and these were more frequent in patients receiving rivaroxaban (13% vs. 4%, HR 3.75; 95% CI, 1.63–8.69%). Survival was similar in both groups [23]. In the third study (ADAM VTE), administration of apixaban (2 × 10 mg for a week then 2 × 5 mg for six months) was compared with dalteparin (200 IU/kg/day for a month, and then 150 IU/kg/day for five months) in 300 patients with CAT. The primary efficacy endpoint for the study was the number of major bleeds that occurred, and no significant differences were found between the two trial arms (0.0% in the apixaban arm and 2.1% in the dalteparin arm; *p* = 0.138). Similarly, the total number of major bleeds and CRNMBs did not differ significantly between the two arms of the study (6.2% vs. 6.3%, respectively, *p* = 0.88). However, the number of VTE recurrences was significantly smaller in the group of patients receiving apixaban in comparison to the group treated with dalteparin (0.7% vs. 6.3%, respectively, *p* = 0.02). Mortality for both groups was similar [24]. A meta-analysis of the above studies has recently been published that confirms the reduced risk of VTE recurrence in cancer patients receiving DOAC as compared to dalteparin, although this is at the cost of an increased number of episodes of major bleeding [25]. In recent days, the results of the Caravaggio study, which is an extension of the ADAM-VTE study, were published and included 1155 cancer patients with newly diagnosed thrombosis who were randomly assigned to one of two arms identical to those found in the ADAM-VTE study. The primary endpoint of this study was the number of CAT relapses over a six-month period. No differences were found between the study drugs (5.6% for the apixaban arm and 7.9% for the dalteparin arm, *p* < 0.001 for noninferiority and *p* = 0.09 for superiority). Interestingly, in the subgroup analysis, in patients less than 65 years of age, apixaban was more effective than dalteparin in preventing the recurrence of venous thromboembolism, and its effectiveness decreased inversely in proportion to the age of the patients. As in other studies, the separation of curves describing the number of CAT relapses over time occurs after a month in keeping with a decrease in the dose of dalteparin. There were also no differences in the main safety point, which was major bleeding, observed in 3.8% of patients receiving apixaban compared to 4.0% of patients in the control group (*p* = 0.6). Importantly, this also concerned major gastrointestinal bleeding. In terms of absolute numbers, CRNMB episodes occurred more frequently after apixaban (9.0% vs. 6.0%, respectively). However, total mortality was similar in both groups [26]. The results of the studies referred to above are summarized in Table 1.

Primary or metastatic lesions of the CNS (*central nervous system*) are associated with an increased risk of thrombosis, while at the same time there is an increased risk of intracranial hemorrhage in such patients. In practice, tumor lesions in the CNS are not an absolute contraindication to anticoagulant therapy, and LMWH is used to treat thrombosis in these patients.

Patients with neoplastic lesions of the CNS were not included in the studies described above, or they constituted only a marginal percentage of subjects. One single comparative cohort study has been performed in which a retrospective analysis of patients with primary or metastatic cancer of the CNS, in whom thrombosis occurred, was carried out. Increased risk of intracranial hemorrhage among patients receiving DOAC compared to patients treated with LMWH was not found. Moreover, much less severe bleeding was observed after DOAC treatment in the subgroup of patients with primary CNS tumors [27].

## 3. The Use of DOAC in the Primary Prevention of CAT

Current guidelines recommend the primary prevention of VTE in patients with cancer during hospitalization [28]. However, it is not a standard procedure in outpatient settings, remaining an option in selected patients at high risk of CAT, which can be estimated using prognostic scales such as the well validated Khorana scale (see Table 2) [29]. The usefulness of DOAC in primary CAT prevention has been assessed in two large clinical trials. In the CASSINI study, the effectiveness of rivaroxaban used for a period of six months at a standard dose (10 mg per day) was compared to placebo for the prophylaxis of VTE in cancer patients starting systematic outpatient treatment. A total of 841 patients in whom the Khorana score was 2 or more at baseline were entered into the study, after the exclusion of patients with primary or metastatic CNS lesions due to the significantly increased risk of bleeding complications in these patients. In addition, during the process of selection for the study, patients underwent ultrasound assessment for asymptomatic deep vein thrombosis in order that those with positive results be excluded from the study (4.5% of patients originally qualified to take part). The primary efficacy endpoint for the study was the composite of symptomatic or asymptomatic proximal deep vein thrombosis of the lower limbs, symptomatic deep vein thrombosis of the upper limb or distal part of the lower limb, symptomatic or asymptomatic pulmonary embolism, or VTE-related death, which was objectively assessed by an independent clinical endpoint committee. These events occurred during six months of follow-up in 6.0% of patients receiving rivaroxaban compared to 8.8% of patients in the placebo control group (HR 0.66, 95% CI: 0.40–1.09; *p* = 0.10). There were no differences in the number of complications, both in terms of major bleeding (2.7% in the study group vs. 2.0% in the control group; HR 1.96; 95% CI, 0.59–6.49) and CRNMB (respectively: 2.7% vs. 2.0%; HR 1.34; 95% CI, 0.54–3.32) [30]. The study thus confirmed the safety of rivaroxaban in a selected population of patients with cancer, while demonstrating no effect of such prophylaxis on reducing the incidence of CAT compared to placebo in these patients. It is worth noting that 47% of patients enrolled in the study prematurely terminated participation while remaining under observation, which had a significant impact on the results obtained, as nearly 40% of events related to the primary endpoint occurred in this group of patients. Additional analysis, including observation limited to the treatment period, showed a statistically significant reduction in the number of primary endpoint events in the rivaroxaban arm of the study (6.4% vs. 2.6%; HR 0.40, 95% CI, 0.20–0.80).

Different results were obtained in the AVERT study that included 574 patients with cancer who were initiating systemic outpatient treatment and in whom, as in the CASSINI study, the risk of VTE was assessed on the Khorana scale with a cut-off of at least 2 points. Patients with myeloproliferative neoplasms, acute leukemias, and those at increased risk of major bleeding, such as occurring in liver disease associated with coagulopathy, were excluded from the study. Patients were assigned to one of two study arms in which they received apixaban 2.5 mg twice daily for six months or a placebo in the control arm. The primary endpoint was defined as the percentage of confirmed VTE (defined as symptomatic or asymptomatic proximal deep vein thrombosis in the lower and upper extremities, pulmonary embolism, or death due to pulmonary embolism) within six months of randomization. It is worth noting that unlike in the CASSINI study, no routinely repeated ultrasound examinations were performed. Statistically, apixaban significantly reduced the incidence of VTE (4.2% vs. 10.2%, HR 0.41; 95% CI: 0.26–0.65; *p* < 0.001) compared to placebo. At the same time, major bleeding was more frequently observed in the apixaban arm (3.5% vs. 1.8%, HR 2.00, 95% CI: 1.01–3.95) [31]. While the overall mortality in the above studies was similar in relation to the study arms, it differed significantly between the two studies. This is most likely due to differences in the types of cancer included in the two studies, in the first of which, half of the patients were being treated for pancreatic or stomach cancer, while in the second, patients with lymphomas or gynecological cancers predominated. Significant differences in the observed frequency of thrombosis and hemorrhagic complications between the two studies can be explained in terms of the design, course, and analysis (on-treatment analysis and intention-to-treat analysis) of both studies. Firstly, the initial screening test for patients with VTE in the CASSINI study likely led to a reduction in the number of events during the study, and these individuals were not included in the analysis (4.5% of the patients originally qualified to participate). Secondly, the longer average period of pharmacotherapy among patients in the AVERT trial may have contributed to the increased number of complications. The most relevant data that support use of DOAC have been extracted from both studies in Table 3.

## 4. Limitations of Direct Oral Anticoagulant Therapy

The wide therapeutic window for DOAC makes it possible to achieve the correct therapeutic plasma concentrations in people weighing 40–120 kg using the same standard dose. Due to the etiology of tumors as well as their impact on metabolism, there is an increased likelihood of weight disorders among patients with malignant tumors. It is estimated that obesity is currently one of the main causes of cancer [32], and hence obese people constitute a significant percentage of patients with malignant tumors. In these patients, however, the use of DOAC may not be sufficiently clinically effective; moreover, data on the effectiveness of DOAC in this group of patients are lacking. At the opposite end of the spectrum, over the course of cancer, some patients suffer from extreme cachexia. For people severely debilitated in this way, the use of DOAC may increase the risk of hemorrhagic complications. Hence, LMWH would appear to be a better choice in the treatment/prevention of CAT in people with significant weight disorders.

Interactions with other drugs may be problematic during DOAC therapy. All DOACs are transported by P-glycoprotein, and in addition, rivaroxaban and apixaban are substrates for cytochrome P450 (CYP3A4) [33]. Many drugs used in systematic anticancer therapy and adjunctive therapy are inhibitors or inducers of P-glycoprotein and/or CYP3A4, which may potentially result in a change in plasma DOAC concentration, taking it outside the therapeutic window. The consequence of this may be lack of a therapeutic effect or an increase in the number of bleeding complications [34]. Despite the fact that any direct interactions between DOAC and oncological drugs have not been evaluated so far, in patients qualifying for DOAC therapy, it is necessary to take into account the systemic treatments used that include both classic cytostatics and drugs used in hormone therapy, targeted therapy, and supportive therapy. In addition, some oncological surgery and radiation therapy may affect DOAC absorption, thereby interfering with therapeutic concentrations [35]. Drugs used in the treatment of cancer patients that have known effects on CYP3A4 and/or P-glycoprotein, and consequently affect the pharmacokinetics of DOAC, are summarized in Table 4 [36,37].

Furthermore, renal impairment limits the use of DOAC. Most clinical trials assessing the usefulness of DOAC in VTE excluded patients with creatinine clearance below 30 mL/min (for apixaban: below 25 mL/min) and DOAC should not be used in these patients. However, no data are available on the appropriate management of patients with less severe renal dysfunction. The only available dose reduction recommendations during VTE therapy are for edoxaban, which requires a half dose reduction in patients with creatinine clearance between 30 and 50 mL/min [22]. Despite the favorable DOAC safety profile demonstrated in studies in patients without active cancer [38], bleeding complications are more common in patients with cancer. A detailed analysis of hemorrhagic complications in the HOKUSAI VTE study has provided interesting information. Major bleeding occurred mainly in the upper gastrointestinal tract (56.2% of all major bleeds in the edoxaban arm compared to 18.8% in the dalteparin arm). Bleeding was most commonly observed in patients with gastrointestinal cancer, among whom 12.7% experienced major bleeding during treatment with DOAC compared to 3.6% of patients treated with LMWH (a statistically significant difference). Significant differences in the frequency of major bleeding between these trial arms also occurred in patients with genitourinary cancers (4.3% vs. 1.4%), especially bladder cancers (12.5% vs. 0.0%) [39]. Somewhat different data are provided by the Caravaggio study in which no increase in major bleeding was observed among patients with gastrointestinal malignancies. At present, due to conflicting data, the use of DOAC for patients with gastrointestinal or urological malignancies would appear risky. Based on the findings of the Caravaggio study, it seems that apixaban is the safest of the DOAC medications; however, caution has to be exercised with regard to possible bleeding complications with apixaban probably being the safest in this group of patients.

Central vein catheters and vascular ports are increasingly implanted during systemic therapy. The presence of a catheter in cancer patients predisposes them to thrombosis in the veins of the upper extremities. Although no direct comparison has been made between the various anticoagulants in the treatment of VTE in these patients, LMWH remains the standard. A small prospective study evaluated the efficacy of rivaroxaban in 70 cancer patients with VTE associated with central venous catheter placement. The large number of bleeding complications occurring during 12 weeks of treatment (12.9%), and the occurrence of fatal pulmonary embolism call into question the safety of DOAC in patients with a diagnosis of cancer [40].

Thrombocytopenia is one of the factors affecting the individual risk of bleeding. At the same time, thrombocytopenia does not reduce the number of VTE recurrences. DOAC therapy in patients with malignant tumors can be safely carried out with a platelet count above 50 G/L. In patients with a platelet count below 50 G/L, DOAC should be discontinued in favor of LMWH. Further anticoagulation therapy during periods of severe thrombocytopenia should be carried out in accordance with LMWH guidelines.

The optimal duration of CAT anticoagulation therapy is unknown. The risk of recurrence of VTE due to active oncological disease still exists even after six months from the first VTE episode [41]. Data from studies on the use of LMWH in patients with cancer confirm the validity and safety of prolonged anticoagulant therapy, with the number of episodes of major bleeding dropping significantly after six months of treatment [42,43]. The safety of prolonged treatment with DOAC is indirectly confirmed by the HOKUSAI VTE study in which the period of active therapy included periods of up to 12 months. An ongoing study (API-CAT, NCT03692065) is currently assessing the benefits of full vs. reduced dose apixaban at the end of six months of standard CAT therapy. To sum up, despite limited evidence, prolonged DOAC therapy in CAT treatment seems justified, which is partly extrapolated from studies involving LMWH.

Bearing in mind that DOAC is absorbed in the gastrointestinal tract, concerns about the pharmacokinetics of this group of drugs in patients after oncological surgery of the gastrointestinal tract or in other disorders that reduce the absorbent surface of the gut seem justified. No data are available on the characteristics of individual DOAC medicinal products in this regard, and the available literature is limited to case reports [44]. Therefore, caution in these patients would seem justified.

**Figure 1:**
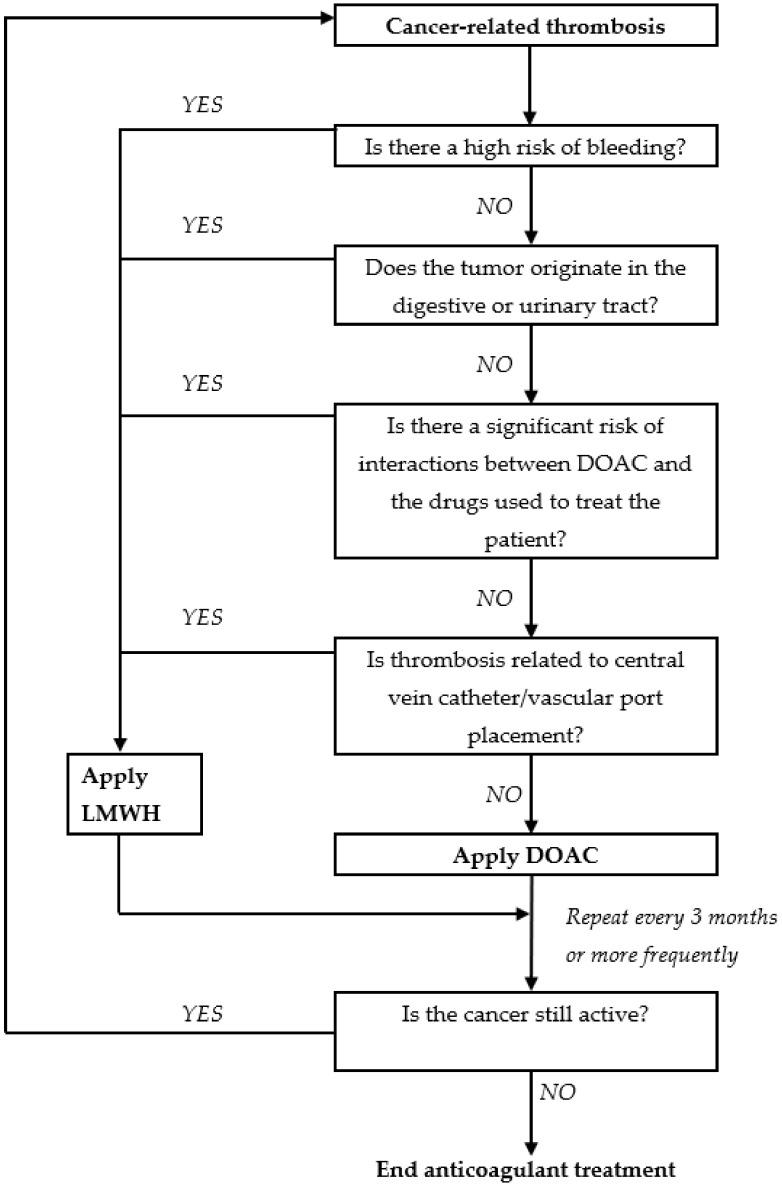
A proposed algorithm for facilitating a safe choice between direct oral anticoagulants and low molecular weight heparin for thrombosis treatment in patients with malignant tumors. Adapted from Suryanarayan, 2019 [43,44].

The above limitations mean that the use of DOAC for the treatment of VTE in patients with cancer requires appropriate selection of patients. The following algorithm, proposed by Suryanarayan [44,45] and modified by the authors, takes into account the specificity of this group of drugs and would appear to enable the appropriate selection of patients for treatment using DOAC (Figure 1).

## 5. Patient Preferences and the Route of Administration of Anticoagulants

From the physician’s perspective, patients’ preferences in choosing the route of drug administration are unequivocal: oral anticoagulants are superior to LMWH in this respect. Adherence to medical recommendations has a significant impact on the effectiveness of treatment, including successful anticoagulation. Therefore, in terms of patients’ preferences that take into account such factors as fear of injections, the choice of therapy may be important. However, in this respect most patients with malignant tumors express a viewpoint that is contrary to expectations. A study was conducted in which 100 oncology patients being treated for CAT were asked to assess the most important attributes of anticoagulants (LMWH or VKA), after being given specific information in this regard. The results revealed that the greatest concern to patients was that anticoagulant therapy should not interfere with cancer treatment (39%), which suggests that cancer is perceived as more important than VTE despite the inherent risk of the latter. The reduction of thrombosis recurrence (24%) was in second place, followed by low risk of bleeding (19%). The superiority of oral administration over injections (13%) was ranked in fourth place [46]. On the other hand, quality of life (QoL) analyses conducted in the ADAM study showed significantly better results among those receiving oral anticoagulants compared to those receiving them in the form of injection. The discomfort associated with daily injections may have an impact on compliance with medical recommendations, and this may have caused the difference in the median duration of treatment between the arms of the HOKUSAI study (211 days vs. 184 days; *p* = 0.01). Nevertheless, the authors believe that from the perspective of an oncological patient, the choice between the use of DOAC and LMWH would not be significantly affected by the route of drug administration.

## 6. Anticoagulation Therapy and the Use of DOAC in Hospice patients

After exhausting the possibilities for causal treatment in patients with advanced malignancy, symptomatic treatment is required in most cases. As the disease progresses and end-of-life care becomes a necessity, for some patients such treatment is delivered in the form of hospice care. The occurrence of CAT in this group of patients, who are frequently in a poor general condition and have additional comorbidities, is a further challenge. The incidence of VTE in patients receiving palliative care remains unknown. However, as the risk of VTE increases with the progression of oncological disease, hospitalization, dehydration, and prolonged immobilization, the increased risk for CAT is also likely to affect patients in hospice care [47,48]. Focusing hospice treatment on maintaining the highest QoL, even at the expense of prolonging it, is an additional problem in the event of symptomatic CAT, which may be manifested by limb or chest pain, shortness of breath, and mental suffering. The occurrence of these symptoms significantly worsens QoL, which should be taken into account when making therapeutic decisions [49]. Guidelines for CAT treatment recommend anticoagulant therapy without specifying its duration in patients with active cancer, and there are no guidelines to define its legitimacy in end-of-life management [50]. Analysis of a series of 214 cases of patients with CAT who died during a two-year follow-up, found that treatment was continued until death in half of the patients, while in 11% it was discontinued ≤7 days before death (patients treated with LMWH). Patients whose anticoagulant treatment was terminated did not have any recurrence of previous CAT symptoms. However, CRNMB was observed in 7% of patients in whom anticoagulant therapy was continued until death [51]. Another observational study involving nearly 1200 patients admitted to palliative care wards, 90% of whom had malignancies, demonstrated a low incidence of VTE. In nearly 10% of patients clinically significant bleeding was observed and was associated primarily with prophylactic anticoagulation [52]. On the basis of these data, LMWH anticoagulation prophylaxis in hospice patients would seem to be inadvisable. There have been no studies dedicated to the use of DOAC in patients with cancer at the end of life. Furthermore, the large randomized DOAC studies in cancer patients described previously did not include patients with a life expectancy of less than six months. It is also worth noting that organ dysfunction, polypharmacy, and cachexia are common among hospice patients, and this may affect the safety of DOAC therapy. These considerations lead to the conclusion that DOAC should not currently be used in this patient population, and in the event of deteriorating QoL symptomatic of CAT, the use of LMWH should remain the treatment of choice.

## 7. The Effect of DOAC on Tumor Growth and Metastatic Dissemination in Experimental Models

The two-way relationship between coagulation and cancer is well known. Preclinical studies, mainly in animal models where drugs were applied before inoculation with cancer cells, have demonstrated the influence of VKA and LMVH on reducing tumor growth and inhibiting the formation of metastases [53,54]. The increasing role of DOAC in the treatment of cancer patients has prompted researchers to conduct experimental studies that assess the effects of DOAC on cancer biology [55]. Most of the available data relate to drugs currently not in clinical use with cancer patients.

The carcinogenicity of ximelagatran was assessed by comparing mice given the drug for 18 months to a control group. Pancreatic cell hyperplasia discovered in individual mice on post-mortem examination prompted the researchers to continue the study in rats, among which, a selected group received a particularly high daily dose of ximelagatran for 24 months. In this group, the development of hyperplastic pancreatic tissue, adenomas or cancer of the pancreas were observed among male rats. There were no changes in other organs. This may indicate a dose- and time-dependent effect on carcinogenesis [56]. Another study demonstrated an increase in lung metastases in mice vaccinated with melanoma cells and treated with ximelagatran prior to inoculation. However, somewhat different results were provided by a study in which female mice were injected with breast cancer cells and administration of dabigatran was started concurrently. In the following weeks, a decrease in tumor volume was observed and there was a tendency for metastatic changes in the lungs and liver to decrease [57]. By contrast, however, in two experiments, Alexander et al. did not observe reduction of the primary tumor [58] nor any effect on metastatic changes [59] in mice during the administration of dabigatran, initiated during the development of the neoplastic changes. Thus, the effect of dabigatran on tumor reduction appears to be negligible for already established tumors. Moreover, in an experiment in which mice were vaccinated with pancreatic cancer cells and dabigatran was started after one week, an increase in tumor dissemination was observed in comparison to the control group. Researchers explained this in terms of increased bleeding within the tumor [60]. Interesting results have been provided by studies using rivaroxaban. In the first of these, fibrosarcoma cells were injected into mice, and after 14 days the animals were randomized for rivaroxaban treatment. A reduction of approximately 50% in tumor mass and a significant reduction in lung metastases was observed. In a further experiment, when randomization was carried out at a later point in time, the effect of rivaroxaban on tumor size was smaller. Similar data were obtained in additional experiments using colorectal and breast cancer models [61]. The effect of rivaroxaban on tumor growth or tumor cell proliferation, in turn, has not been confirmed in pancreatic cancer or triple-negative breast cancer experimental models in immunodeficient mice [62,63]. The above data suggest that the role of DOAC in cancer biology is uncertain and requires further research.

## 8. Conclusions

Direct oral anticoagulants provide an attractive alternative to LMWH in the treatment of VTE in cancer patients. Studies have confirmed both the efficacy and safety of this group of drugs in the treatment of CAT. However, the limitations of DOAC associated with an increased risk of major bleeding, interaction with other drugs, unknown or inappropriate pharmacokinetics in patients with large deviations from normal body weight, and in patients with impaired renal function means that CAT therapy using DOAC requires patients to be carefully selected for this form of treatment. Currently, DOAC in CAT treatment is an alternative to LMWH in the recommendations of some scientific societies, including the National Comprehensive Cancer Network (NCCN) and the International Society on Thrombosis and Haemostasis (ISTH) [64,65]. Direct oral anticoagulants have also been included in the recently published American Society of Clinical Oncology (ASCO) recommendations: supporting the use of rivaroxaban in initial anticoagulant therapy and in combination with edoxaban in prolonged therapy. These recommendations are based on the strength and quality of the evidence available [66]. Furthermore, data obtained from studies assessing DOAC in the primary prevention of CAT in high-risk patients are very encouraging, which prompted the inclusion of rivaroxaban and apixaban in the latest ASCO and The International Initiative on Thrombosis and Cancer (ITAC) guidelines [67]. Ongoing subsequent phase III studies, as well as data from actual clinical practice, will determine the optimal role of DOAC in cancer patients, both in the treatment and prevention of VTE. However, DOAC is a long-awaited alternative that has irrevocably ended the dominance of LMWH in oncology.

## Figures and Tables

**Table 1:** Data from prospective clinical trials comparing the safety and efficacy of direct oral anticoagulants and low molecular weight heparin in the treatment of thrombosis in cancer patients.

Study	Hokusai Cancer VTE		SELECT-D		ADAM-VTE		Caravaggio	
Patient Population	Adults with cancer and newly diagnosed symptomatic or incidental thrombosis		Adults with cancer and newly diagnosed symptomatic or incidental thrombosis		Adults with cancer and newly diagnosed symptomatic or incidental thrombosis		Adults with cancer and newly diagnosed symptomatic or incidental thrombosis	
Observation time (months)	12		6		6		6	
Anticoagulant	Edoxaban	Dalteparin	Rivaroxaban	Dalteparin	Apixaban	Dalteparin	Apixaban	Dalteparin
Treatment	LMWH for 5 days, then edoxaban 60 mg daily	200 IU/kg/day for 30 days, then 150 IU/kg/day	15 mg twice daily for 3 weeks, then 20 mg once daily	200 IU/kg/day for 1 month, then 150 IU/kg/day	10 mg twice daily for 7 days, then 5 mg twice daily	200 IU/kg/day for 1 month, then 150 IU/kg/day	10 mg twice daily for 7 days, then 5 mg twice daily	200 IU/kg/day for 1 month, then 150 IU/kg/day
Sample size	522	524	203	203	145	142	576	579
Mean age of patients (years)	64.3 (SD = 11)	63.7 (SD = 11.7)	67 (22–87)	67 (34–87)	64.4 (SD = 11.3)	64.0 (SD = 10.8)	67.2 (SD = 11.3)	67.2 (SD = 10.9)
Metastatic disease (%)	52.2	53.4	58	58	65.3	66.0	67.5	68.4
Recurrence of thrombosis (%)	7.9	11.3	4	11	0.7	6.3	5.6	7.9
	HR 0.71, 95% CI 0.48–1.06 *p* = 0.09		HR 0.43, 95% CI 0.19–0.99 *p* = NR		HR 0.099, 95% CI 0.01–0.78 *p* = 0.0281		HR 0.63, 95% CI 0.37–1.07 *p* = 0.09	
Major bleeding (%)	6.9	4	6	4	0	2.1	3.8	4.0
	HR 1.77, 95% CI 1.03–3.04 *p* = 0.04		HR 1.83, 95% CI 0.68–4.96 *p* = NR		*p* = 0.138		HR 0.82, 95% CI 0.40–1.69 *p* = 0.60	
CRNMB (%)	14.6	11.1	13	4	6.2	4.2	9.0	6.0
	HR 1.38, 95% CI 0.98–1.94 *p* = NR		HR 3.76, 95% CI 1.63–8.69 *p* = NR		NR *		HR 1.42, 95% CI 0.88–2.30 *p* = NR	
Mortality (%)	39.5	36.6	25	30	16	11	23.4	26.4
	HR 1.12, 95% CI 0.92–1.37 *p* = NR		NR		HR 1.40, 95% CI 0.82–2.43 *p* = 0.307		HR 0.82, 95% CI 0.62–1.09 *p* = NR	
Median duration of treatment	211 days	184 days	5.9 months	5.8 months	5.78 months	5.65 months	178 days	175 days

CRNMB—clinically relevant non-major bleeding, NR—not reported; * statistics for CRNMB and major bleeding were tested cumulatively. The results of the comparison were statistically insignificant.

**Table 2:** Khorana scale (with modifications by the *American Society of Clinical Oncology*) for assessing the risk of venous thromboembolism in patients receiving chemotherapy in an ambulatory setting.

Clinical Characteristics:	No. of Points:
Type of cancer: stomach, pancreas, primary brain tumors (very high risk) Lungs, lymphoma, reproductive organs, bladder, kidneys (high risk)	2 1
Platelet count prior to chemotherapy ≥350,000/µL	1
Leukocyte count prior to chemotherapy >11,000/µL	1
Concentration of hemoglobin prior to chemotherapy <10 g/dL and/or use of erythropoietin	1
BMI ≥ 35 kg/m^2^	1

Interpretation: 0 points—minimal risk, 1–2 points—medium risk, ≥3 points—high risk.

**Table 3:** Data from prospective clinical trials assessing the efficacy and safety of direct oral anticoagulants in the prevention of thrombosis in cancer patients.

Study	CASSINI		AVERT	
Population	Adult patients starting chemotherapy assessed according to the Khorana scale ≥2 points		Adult patients starting chemotherapy assessed according to the Khorana scale ≥2 points	
Period of observation (months)	6		6	
Anticoagulant	rivaroxaban	placebo	apixaban	placebo
Group size	420	421	291	283
Mean age of patients (years)	63 (23–87)	62 (28–88)	61.2 (SD = 12.4)	61.7 (SD = 11.3)
Type of analysis	on-treatment analysis		intention-to-treat analysis	
Occurrence of thrombosis (%)	2.62	6.41	4.2	10.2
	HR 0.40, 95% CI 0.20–0.80 *p* = 0.007		HR 0.41, 95% CI 0.26–0.65 *p* < 0.001	
Major bleeding (%)	1.98	0.99	3.5	1.8
	HR 1.96, 95% CI 0.59–6.49 *p* = 0.265		HR 2.00, 95% CI 1.01–3.95 *p* = 0.046	
CRNMB (%)	2.72	1.98	7.3	5.5
	HR 1.34, 95% CI 0.54–3.32 *p* = 0.53		HR 1.28, 95% CI 0.89–1.84 *p* = NR	
Mortality (%)	20.0	23.8	12.2	9.8
	HR 0.83, 95% CI 0.62–1.11 *p* = 0.213		HR 1.29, 95% CI 0.98–1.71 *p* = NR	

CRNMB—clinically relevant non-major bleeding, NR—not reported.

**Table 4:** Drugs used in oncological therapy with known effects on cytochrome P450 and/or P-glycoprotein.

Type of Interaction	CYP3A4	P-Glycoprotein
Inducers (may increase DOAC plasma levels)	Cytostatics: paclitaxel, docetaxel, vincristine, vinorelbine Hormonal drugs: **enzalutamide** * Immunomodulators: **dexamethasone**, prednisone	Cytostatics: **vinblastine**, **doxorubicin** Tyrosine kinase inhibitors: **vandetanib**, **sunitinib** Immunomodulators: **dexamethasone**
Inhibitors (may reduce DOAC plasma levels)	Cytostatics: etoposide, doxorubicin, idarubicin, ifosfamide, cyclophosphamide, lomustine Tyrosine kinase inhibitors: imatinib, crizotinib, nilotinib, lapatinib, dasatinib Hormonal drugs: abiraterone, anastrozole Immunomodulators: cyclosporine, tacrolimus, temsirolimus	Tyrosine kinase inhibitors: **imatinib**, **crizotinib**, nilotinib, lapatinib Hormonal drugs: **abiraterone**, **enzalutamide**, **tamoxifen** Immunomodulators: cyclosporine, tacrolimus
Other substrates for CYP3A4 or/and P-glycoprotein	Cytostatics: vinblastine, irinotecan, busulfan Tyrosine kinase inhibitors: vemurafenib, vandetanib, sunitinib, erlotinib, gefitinib Monoclonal antibodies: brentuximab Hormonal drugs: bicalutamide, tamoxifen, flutamide, letrozole, fulvestrant Immunomodulators: everolimus	Cytostatics: paclitaxel, docetaxel, vincristine, vinorelbine, methotrexate, irinotecan, etoposide, daunorubicin, bendamustine

Adapted from Steffel et al., 2018 [37]; * especially strong interactions are printed in bold type.
